# Building Disaster-Ready Research Ecosystems: Evidence from Doctoral Scholars from Tertiary Care Institute in India

**DOI:** 10.14789/ejmj.JMJ25-0030-OA

**Published:** 2025-12-18

**Authors:** ASHWINI A MAHADULE, MEENAKSHI KHAPRE, RANJEETA KUMARI, SHALINEE RAO

**Affiliations:** 1 Department of Physiology, All India Institute of Medical Sciences, Rishikesh, Uttarakhand, India; 1 Department of Physiology, All India Institute of Medical Sciences, Rishikesh, Uttarakhand, India; 2 Department of Community and Family Medicine, All India Institute of Medical Sciences, Rishikesh, Uttarakhand, India; 2 Department of Community and Family Medicine, All India Institute of Medical Sciences, Rishikesh, Uttarakhand, India; 3 Department of Pathology, All India Institute of Medical Sciences, Rishikesh, Uttarakhand, India; 3 Department of Pathology, All India Institute of Medical Sciences, Rishikesh, Uttarakhand, India

**Keywords:** COVID-19 pandemic, coping strategies, disaster preparedness, mixed-method study, research disruption, PhD scholars

## Abstract

**Background:**

Pandemics and natural disasters have historically disrupted research ecosystems worldwide. In India, for example, the COVID-19 epidemic, the 2004 Indian Ocean tsunami, and the 1918 influenza pandemic have not only impacted education systems but also research-related activities. Early-career scholars were disproportionately affected by the severe disruptions to research activity induced by the COVID-19 Pandemic.

**Aim:**

To determine and examine the difficulties PhD research scholars encountered in India during the COVID-19 Pandemic and to understand coping mechanisms used to minimise uncertainties.

**Methods:**

Among full-time PhD scholars at a tertiary healthcare institution in India, we conducted a sequential explanatory mixed-methods study. A pre-validated online questionnaire was used to gather quantitative data, and focus group discussions (FGDs) were held to examine problems and potential solutions further. Descriptive statistics, thematic analysis, and triangulation of questionnaire and FGD results were used to examine the data.

**Results:**

16 scholars participated in FGDs, and 41 scholars completed the questionnaire. Administrative barriers, logistical obstacles, time limits from pandemic-related tasks, participant recruitment issues, infrastructure closures, and missed opportunities for collaboration were the six thematic domains of difficulty that surfaced. Five types of coping techniques were identified: emotion-focused, meaning-focused, problem-focused, problem-avoidance, and social-focused coping. Adaptive measures, such as local material sourcing, skill development through online platforms, and alternative data collection, were used by scholars.

**Conclusion:**

Natural calamities and pandemics have a systemic impact on research, particularly for early-career researchers. Flexible deadlines, emergency funding, streamlined teamwork, and crisis-adaptive ethics review procedures were among the few recommendations.

## Introduction

Large-scale natural disasters and infectious disease outbreaks have repeatedly disrupted research and societal functioning worldwide. In India, the 1918-1919 influenza pandemic was particularly catastrophic, causing an estimated 12-18 million deaths, nearly 6% of the country’s population and revealing how pandemics can overwhelm healthcare systems, paralyze social structures, and halt academic progress^1)^. Since independence, India has experienced numerous sudden-onset disasters, including the 1999 Odisha super-cyclone, the 2001 Gujarat earthquake, the 2004 Indian Ocean tsunami, the 2013 Uttarakhand flash floods, and the 2018 Kerala floods. Each event caused substantial human and infrastructural losses, including damage to educational and research institutions^2-6)^.

In the 21st century, the coronavirus disease 2019 (COVID-19) pandemic created an unprecedented global crisis. Laboratory closures, halted fieldwork, delayed clinical trials, and redirected funding significantly hindered scientific productivity^7, 8)^. Several reports have documented how early-career researchers were disproportionately affected by these disruptions, facing challenges such as loss of access to research facilities, career uncertainty, and increased psychosocial stress^7-9)^.

Disasters influence research continuity through multiple mechanisms like destruction or inaccessibility of laboratories and field sites, diversion of funds and institutional priorities, delayed ethics approvals due to infection-control requirements, reduced participant availability, and heightened caregiving responsibilities, which often impact specific groups more severely^8, 10)^.

The present study builds on this global evidence by examining the unique challenges faced by PhD scholars in India during the COVID-19 pandemic. Using a sequential explanatory mixed-method approach, we aimed to assess the barriers encountered by doctoral scholars and identify coping strategies that enabled them to continue research under constraints. Insights from this study may guide the development of institutional policies and preparedness measures to ensure continuity of research during future crises.

## Materials and Methods

### Study design and setting

The present study was a sequential explanatory mixed-method study. We first assessed the challenges faced by PhD research scholars during the COVID-19 pandemic, followed by a focus group discussion (FGD) to understand the challenges and possible solutions. We included full-time PhD scholars enrolled in the course from the first and second wave of the COVID-19 Pandemic in a tertiary health care institute willing to participate in the study. We obtained informed written consent after explaining the study protocol and procedures. Participants did not receive any incentives or financial compensation to participate in this study. Approval was taken from the institutional ethics committee prior to commencement of the study. (AIIMS/IEC/20/793/21-11-2020)

### Study procedure

Data was collected online using a pre-validated questionnaire (Google form) for further focus group interviews. All research scholars were invited to fill an open-ended questionnaire regarding the effect of a pandemic on PhD research activities.

Further, we invited scholars for focus group discussion through an online mode using the Google Meet platform in a group of 6-8 participants. Invitation for the FGD was sent via email, and a phone call was made to the participants for confirmation. The date and time were scheduled in consensus with the participants. The discussion was initiated by welcoming the participants and informing them about the purpose and ground rules of the discussion. We took the consent of participants for the audio recording of the discussion. We followed the FGD guidelines for conducting the FGD, which was done in a sequential manner through series of questions. Only, after obtaining satisfactory responses to the previous questions, the next question was put forward to the participants. Based on the responses recorded in the Google form, challenges and strategies employed to overcome them were confirmed, discussed, and clarified further. A thorough discussion with the participants and brainstorming was carried out for any unaddressed challenges. Later, participants were asked about solutions or their expectations from the authorities. In the end, the moderator (MK) summarised the discussion and reached a group consensus. This discussion lasted for 45 minutes to an hour for each session. FGDs were carried out until data saturation, when no new codes emerged.

### Data analysis

Data was compiled as percentages/proportions for closed-ended responses. Open coding was used for open-ended responses (verbatim), subsequently categorized. The thematic analysis approach was used to identify the emergent themes. Transcripts of FGD were written on the same day. Data triangulation of Google form and FGD transcripts was carried out to identify the codes. Transcripts were read and re-read by two independent researchers (MK and AM) who independently coded and identified the categories. Third researcher (SR) resolved any discrepancies as required. Findings were shared with participants for accuracy and resonance with their experience.

## Results

**Figure 1:**
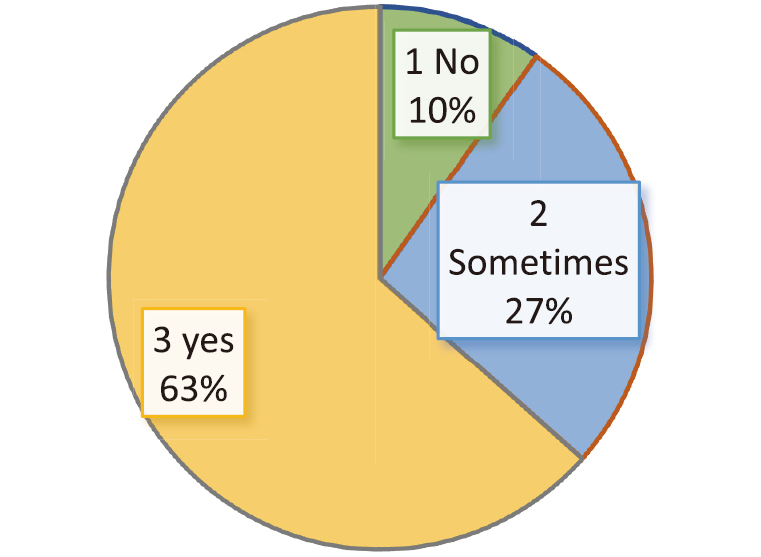
Difficulty in pursuing research work during COVID-19 Pandemic (N = 41)

A total of 41 PhD scholars completed the online questionnaire, and 16 scholars participated in two focus group discussions (FGDs), after which data saturation was achieved. Demographic details are presented in Table 1. The majority of participants were aged 25-30 years, with near-equal gender distribution (49% male and 51% female). Most participants reported experiencing major disruptions to their research activities during the pandemic (Figure 1).

Triangulation of questionnaire and FGD data revealed six key thematic domains of challenges (Table 2):

**1. Administrative barriers**: Prolonged approval processes for guide changes, thesis confirmation, and funding allocations were widely reported. Several participants described delays in fellowship disbursements and ethical reviews, often extending over several months due to shifting institutional priorities.

• “I had applied for a supervisor change in February 2020, but it took seven months due to COVID- related administrative delays.” (26 years)

• "Most funding diverted to COVID-related research so not getting funds for my project" (23 years).

**2. Logistics obstacles**: Supply chain disruptions prevented timely procurement of reagents and consumables.

• “Radioisotopes and radiopharmaceuticals were unavailable for months due to air transport restrictions.” (28 years)

• "A lot of problems like the release of funds as well as supply of reagents were of great concern during this period" (27 years).

**3. Time constraints**: Many scholars were redeployed to pandemic-related clinical or administrative duties, leaving little time for thesis work.

**4. Recruitment challenges**: Hospital restrictions and patient apprehension reduced participant availability for ongoing projects.

• “Fieldwork was interrupted because families did not allow us to interact with participants.” (26 years)

**5. Infrastructure limitations**: Laboratory and library closures halted experimental and literature-based work.

• “For PhD work, we need lab access, but everything was shut, including the library.” (25 years)

**6. Loss of Collaboration**: Travel restrictions and institutional closures disrupted collaborations with other departments or institutions.

• “Our collaborating institute was closed, so I could not perform my data analysis.” (25 years)

• "External Co-guides was not allowing to work in their institute because we were from the medical institute."(25 years)

Further, discussion with the participants focussed on their coping strategies to overcome the delay in their thesis work.

For this purpose, we identified five different coping strategies (Table 2).

**1. Problem focused** - Many adapted their work methods by shifting to online data collection, sourcing local materials, or modifying project designs.

• "I planned to collect data by personally visiting the institutes, but this was not possible, so I decided to collect data online" (24 years).

• "I traced diabetic patient records from the diabetic clinic and received information from diabetic nurse educators as well. With starting of Out Patient Department (OPD) things are much better and hope it will proceed like that" (26 years).

**2. Problem avoidance** - A few scholars deferred activities or changed topics altogether.

• "I could not overcome those challenges due to logistic problems" (25 years).

• "I changed area of research and statement." (32 years)

**3. Emotion-focused coping** - Several expressed optimism and perseverance despite frustrations.

• “It used to take two weeks to get reagents before COVID, now it takes months, but we can only wait and hope.” (32 years)

**4. Meaning-focused**- Some used the downtime for skill enhancement through online workshops and manuscript writing.

• "By turning the pandemic situation into an opportunity for learning new subjects and joined online workshops/webinars for this purpose." (29 years)

• "In the meantime, I have published 1 article in a PubMed-indexed journal, and 2 more articles are under review." (27 years)

**5. Social focussed** - Support from peers, mentors, and family was crucial for maintaining motivation.

“Self-help books and support from friends helped me stay positive.” (26 years)

COVID-19 pandemic has been there for almost one and half years, and research scholars have been trying to find ways to cope with problems. Scholars were asked to give some solutions for such pandemic situations in the future. Scholars recommended several measures for disaster resilience:

• Flexible timelines and lenient reviews for affected thesis submissions.

• Provision of emergency research funds and extended fellowships.

• Simplified inter-institutional collaboration procedures.

• Policy allowances for modified sample sizes or remote data collection during crises.

**Table 1:** Distribution of age(years) among Ph, D. research scholars (N = 41)

Sr. No.	Age group	Percent
1	25-27	31.7
2	28-30	36.6
3	31-33	17
4	34-36	12.2
5	37-39	2.4
Total		100

**Table 2:** Challenges in thesis related work during COVID 19 pandemic and coping style

Theme	Categories
**Reasons for delay of thesis during COVID-19 pandemic**	
Administrative	Change in guide
	Funding got delayed or limited funds
	DRC and IEC delayed
Logistic	Transport issues due to lockdown
Time	Overburdened COVID duties
Collection of data	Difficulty in the recruitment of subjects
	No field work allowed
Infrastructure	Laboratory/ library was closed
Collaboration	Collaboration not done with other departments
	Difficulty in collaboration with other Institute
**Coping strategies (Coping Circumplex Model (CCM))**	
Problem-focused coping	Working overtime
	Efforts to buy logistics
	An alternative way for data collection
	Put own expenses to complete work
Problem avoidance	Changed thesis topic
	Accepted situation without giving a try
Emotion focus coping	Hope
	Helplessness
Meaning focused	Published paper / attended seminars, webinars, and improved skills
Social focussed	Help from books, friends

DRC: Doctoral research committee; IEC: Institute ethics committee

## Discussion

The present study explored the multifaceted challenges experienced by PhD scholars during the first and second waves of the COVID-19 pandemic in a tertiary care setting in India and identified the coping strategies they employed to mitigate these disruptions. The findings demonstrate that research progress was hindered across six principal domains—administrative delays, logistical barriers, time constraints due to pandemic-related duties, recruitment challenges, infrastructure closures, and loss of collaborative opportunities. These findings align with global evidence that emphasizes how pandemics and natural disasters impose systemic disruptions on research ecosystems, particularly for early- career investigators^7-11)^.

Globally, disasters have repeatedly demonstrated their capacity to disrupt scientific progress. The 1918 influenza pandemic not only caused devastating mortality but also dismantled data collection systems, undermined ongoing research, and diverted public health resources for years^12)^. Similarly, the 2004 Indian Ocean tsunami, the 2010 Haiti earthquake, and the 2011 Tōhoku earthquake in Japan illustrate how sudden-onset disasters can lead to loss of samples, archives, and laboratory infrastructure, while simultaneously redirecting institutional funding and personnel to immediate humanitarian needs^13-15)^.

Studies following the 2013 Uttarakhand floods and 2001 Gujarat earthquake revealed that disruptions to population cohorts and data continuity led to biased samples and limited follow-up, highlighting the long-term scientific consequences of disasters^14, 16)^. The ethical reprioritization like shift to emergency treatments and surveillance, infection-control limitations that restrict in-person data collecting, and the reallocation of resources and staff to epidemic response are some of the unique challenges that pandemics bring^17)^. The COVID-19 pandemic represented an unprecedented global disruption, halting laboratory work, delaying clinical trials, and impeding academic mobility. Surveys of researchers across Europe, the United States, and Asia reported similar patterns of productivity loss, funding redirection, and widening gender inequities, with women and early-career scholars disproportionately affected^11, 18)-19)^. The present study’s findings echo these trends in the Indian context, where scholars faced administrative gridlocks, funding delays, and institutional closures, emphasizing the vulnerability of developing research ecosystems during crises.

Pandemics compel methodological compromises and ethical recalibrations. Interruptions in participant access, shortened follow-up durations, and the shift toward remote data collection can compromise data validity^17, 18)^. Moreover, infection control and biosafety regulations may delay ethics approvals and data collection. The Nipah virus outbreaks in Kerala and Ebola-related research in Africa demonstrated the ethical tension between urgent research and participant protection^20-22)^. However, such crises also accelerated innovation through digital consent processes, online focus groups, virtual collaboration platforms, and expedited ethics review mechanisms^17, 20)^. Our study’s evidence of adaptive responses such as online data collection and local sourcing of materials illustrates how scholars can convert disruption into opportunity when institutional support exists. Thus from our study we also can deduce some recommendations for preparedness of disaster ready situations and reinforces the urgent need for disaster-ready research ecosystems. Institutional preparedness should include:

1. **Crisis-contingent research continuity plans**, including safe data backups and off-site storage of materials.

2. **Flexible funding and emergency fellowships** for early-career researchers.

3. **Pre-approved rapid ethics review protocols** for public health emergencies.

4. **Investment in hybrid and digital research infrastructure** to ensure uninterrupted data collection.

5. **Cross-institutional collaborations** with simplified administrative procedures.

6. **Mental health and mentorship support** to mitigate burnout during prolonged crises.

These recommendations are consistent with frameworks proposed by the World Health Organisation (WHO) and UNESCO for resilient research systems that sustain inquiry during emergencies while upholding ethical and scientific integrity^23, 24)^.

## Limitations and future directions

The study was limited by its single-institution scope and modest sample size; therefore, findings may not be generalizable to all doctoral programs across India. Future multi-institutional or longitudinal studies could assess the cumulative impact of disruptions on publication productivity, grant timelines, and career progression. Comparative analyses between institutions with differing resource levels may reveal scalable, cost-effective resilience strategies suitable for the Indian context.

## Conclusion

In conclusion, pandemics and natural disasters are not only humanitarian crises but systemic shocks to the research ecosystem. Preparing Indian research institutions to anticipate and adapt to such disruptions through flexible policies, ethical agility, and digital innovation is crucial to safeguard scientific productivity and ensure research continuity during future crises.

## Author contributions

AM- Drafting of manuscript and acquisition of data, MK - Data acquisition and analysis, RK - Critical appraisal and intellectual inputs, SR - Concept and design of study, revising and critical appraisal of manuscript.

## Conflicts of interest statement

The authors declare that there are no conflicts of interest.
